# Postural Control and Upper-Extremity Motor Impairment Associated with Functional Independence During Acute Stroke Hospitalization: A Cross-Sectional Study

**DOI:** 10.3390/healthcare14203362

**Published:** 2026-10-09

**Authors:** Teuta Osmani-Vllasolli, Valbona Govori, Artan Murati, Besfort Kryeziu

**Affiliations:** 1 Faculty of Medicine, University of Prishtina, 10000 Prishtina, Kosovo; teuta.osmani@uni-pr.edu (T.O.-V.); besfort.kryeziu@uni-pr.edu (B.K.); 2 Clinic of Physical Medicine and Rehabilitation, University Clinical Centre of Kosova, 10000 Prishtina, Kosovo; 3 Clinic of Neurology, University Clinical Centre of Kosova, 10000 Prishtina, Kosovo

**Keywords:** postural control, upper extremity, Fugl-Meyer Assessment, functional independence, stroke rehabilitation

## Abstract

*Background and aim:* Postural control and limb motor impairment are essential to functional status after stroke. We aimed to examine the associations of postural control and motor impairment with functional independence during acute stroke hospitalization, with particular attention to the relationship between postural control and upper-extremity motor impairment and whether these domains provide information regarding functional independence. *Methods:* In this single-center cross-sectional study, 51 adults with acute stroke were assessed 3–10 days after onset. Postural control, motor impairment, functional independence, and disability were evaluated using the Postural Assessment Scale for Stroke Patients (PASS), Fugl-Meyer Assessment (FMA), Barthel Index, and modified Rankin Scale (mRS), respectively. Associations were examined using Spearman correlations and multivariable regression. We separately examined the upper-extremity motor impairment using the FMA-UE, and an additional hierarchical regression model evaluated whether PASS provided explanatory information beyond FMA-UE. *Results:* We found that PASS was strongly associated with FMA-UE (ρ = 0.749, 95% CI 0.554–0.881; *p* < 0.001), while FMA-UE was associated with the Barthel Index (ρ = 0.799, *p* < 0.001). A model including age, assessment timing, and FMA-UE explained 83.8% of Barthel Index variance. The addition of PASS increased explained variance to 95.2% (ΔR^2^ = 0.114; *p* < 0.001), with both FMA-UE (B = 0.505, 95% CI 0.296–0.713; *p* < 0.001) and PASS (B = 1.955, 95% CI 1.469–2.441; *p* < 0.001) remaining independently associated with functional independence. *Conclusions:* Postural control and upper-extremity motor impairment were strongly related but provided complementary information regarding functional independence. Integrating postural assessment with upper-extremity motor assessment may improve early functional characterization and rehabilitation planning after stroke.

## 1. Introduction

Worldwide, stroke remains a major cause of mortality, disability, and loss of functional independence. In 2021, an estimated 11.9 million new cases with acute stroke were reported globally, whilst 93.8 million people were living with the consequences of stroke, and stroke was attributed for approximately 7.3 million deaths and 160.5 million disability-adjusted life-years [1]. Several regions have already seen a decline in age-standardized rates of stroke, but the absolute burden still continues to rise, increasing the need not only for effective acute treatment but also for early identification of functional impairment and rehabilitation needs.

The functional consequences of stroke are multidimensional. Weakness, impaired functional motor control, aberrant movement synergies, sensory deficiencies, poor coordination, and postural imbalance may all contribute to dependence in basic activities of daily living. But motor impairment and functional independence are not equivalent, because a patient may retain voluntary movement but remain dependent because of poor sitting or standing control, while another may compensate for persistent limb weakness and preserve some degree of independence. This differentiation is consistent with the International Classification of Functioning, Disability and Health, which separates impairments of body functions from limitations in activity and participation [2]. Accordingly, assessment of stroke disability should extend beyond limb strength or movement alone.

Postural control is especially relevant during acute hospitalization because it supports transfers, sitting, standing, and mobility. Impairments in trunk stability, weight distribution, vertical orientation, and postural reactions can markedly limit self-care even when limb motor function is partly preserved. The Fugl-Meyer Assessment (FMA) is one of the most widely used measures of post-stroke sensorimotor impairment and provides separate upper- and lower-extremity motor scores as well as a combined motor score [3]. In contrast, the Postural Assessment Scale for Stroke Patients (PASS), was designed specifically to capture the ability to maintain and change posture across a wide range of stroke severity, including patients with poor standing ability [4,5]. Then, the functional independence can be evaluated at the activity level using the Barthel Index, which quantifies assistance required for feeding, dressing, hygiene, transfers, mobility, continence, and stair use [6,7]. Strong correlations between PASS, FMA, and Barthel scores may reflect the fact that more severely affected patients perform poorly across all domains.

Upper-extremity motor impairment represents an important component of post-stroke disability because voluntary arm and hand control is required for feeding, combing, dressing, human manipulation, and other daily activities. However, upper-extremity performance does not occur independently of the postural system. Effective trunk stability provides a proximal base for positioning, reaching, and purposeful upper-limb movement, while impaired postural adjustments after stroke may increase corrective trunk movement and restrict the translation of residual upper-extremity motor capacity into functional activity. Additionally, lower-extremity motor function contributes to functional independence through its role in standing, transfers, walking, and stair use [7,8]. Weakness and impaired movement control usually restrict these activities, particularly when accompanied by reduced balance or trunk stability [9,10]. Studies in chronic stroke have linked lower-extremity FMA performance with balance and mobility [8,9]. These functional demands also overlap with upper-extremity activity, as transfers and some self-care tasks require coordinated lower-limb support, trunk stability, and arm use [10,11]. All things considered, these relationships provide a rationale for examining both upper- and lower-extremity motor impairment alongside postural control when assessing functional independence during acute stroke hospitalization.

Studies from the existing literature have examined these relationships using several methodological approaches. A study assessed early trunk control using selected PASS items and applied multivariable stepwise regression to predict comprehensive activities of daily living at six months, considering limb motor impairment and other clinical characteristics [12]. Similarly, another study used the Trunk Impairment Scale (TIS) and forward stepwise regression to predict six-month Barthel Index outcomes in a multicenter rehabilitation cohort [13]. On the other hand, Wang et al. used Rasch analyses to develop a shorter functional assessment combining items from the FMA, PASS, and Barthel Index [14]. Jansa et al. also evaluated the Extended Barthel Index alongside the standard Barthel Index and FMA in patients with acute ischemic stroke, examining its reliability, validity, and responsiveness over the first six weeks after onset [15]. More directly relevant to acute hospitalization, Iso et al. examined PASS, the upper-extremity FMA motor score (FMA-UE), and the Functional Independence Measure during the first week after cerebral infarction using Spearman correlations and stepwise multiple regression; PASS and age were retained in the final model [11]. More recent investigations have used multiple regression to examine relationships of trunk and extremity function with activities of daily living, balance, and gait in chronic stroke [10], and correlation analyses to examine trunk control, sitting balance, and upper-extremity function in subacute stroke [16]. We need further evidence on the additional value of PASS in explaining concurrent Barthel Index scores beyond limb motor impairment during acute stroke hospitalization [11,12,13]. This study addresses this question using separate models adjusted for age, assessment timing, and either upper-extremity or overall limb motor impairment.

Together, these studies support a close relationship among postural control, limb motor function, and daily activities, while addressing different assessment instruments, recovery stages, and analytical questions [10,11,12,13,14,16]. However, these studies do not directly quantify the additional variance in concurrent Barthel Index scores explained by PASS in separate models retaining age, assessment timing, and either upper-extremity or overall limb motor impairment during acute hospitalization. Further examination of this specific question may clarify the complementary information obtained from combined postural and motor assessment.

Also, these studies and findings are particularly relevant for the context of Kosovo, where cerebrovascular diseases remain a leading cause of mortality. Also, even in the regional scale, locally published evidence on acute post-stroke disability and rehabilitation needs remains scarce [17]. Over the past few years, national health policy has increasingly emphasized organized stroke care, service quality, and improved health information through the Stroke Action Plan 2022–2025 and the Health Sector Strategy 2025–2030 [18,19]. In this context, standardized bedside tools that are inexpensive, reproducible, and applicable during routine hospital care may help improve functional documentation and support earlier rehabilitation planning.

Therefore, this study objective was to examine the associations of postural control, measured using PASS, and limb motor impairment, measured using FMA, with functional independence assessed using the Barthel Index and to determine whether postural control provides additional explanatory information beyond limb motor impairment among patients evaluated during acute stroke hospitalization in a tertiary clinic in Kosovo. Using linear regression, we examined whether PASS provided additional explanatory information beyond age, time from stroke onset to assessment, and either FMA-UE or total FMA motor scores. We additionally examined relationships with global disability and associations involving PASS subdomains and upper- and lower-extremity motor performance. These analyses aimed to clarify the information provided by postural and motor assessments and contribute evidence relevant to early functional assessment in Kosovo.

The final structure of the manuscript is organized as follows. Section 2 describes the study population, assessment instruments, and statistical methods. Section 3 presents the results. Section 4 discusses the findings, their clinical relevance, and the strengths and limitations of the study. Section 5 presents the conclusions and directions for future recommended research.

## 2. Methodology

### 2.1. Study Design and Settings

We conducted a single-center cross-sectional observational study within the Clinic of Neurology and Clinic of Physical Medicine and Rehabilitation, University Clinical Centre of Kosova (UCCK), between April to July, 2026. In this study, we evaluated motor impairment, postural control, and functional independence at a single time point during among patients hospitalized after acute stroke. Each patient contributed one set of clinical and functional assessments. All standardized assessments were performed during that admission, within 24 h of admission to the participating clinical unit, after the treating team considered the patient clinically stable. The interval from stroke onset to assessment was recorded separately and ranged from 3 to 10 days. Thus, the 24 h window refers to the interval between clinical-unit admission and assessment, whereas the 3 to 10-day interval refers to the time elapsed between stroke onset and the same assessment. The reporting of this study was reviewed against the Strengthening the Reporting of Observational Studies in Epidemiology (STROBE) recommendations for cross-sectional studies [20].

### 2.2. Study Population

**Figure 1:**
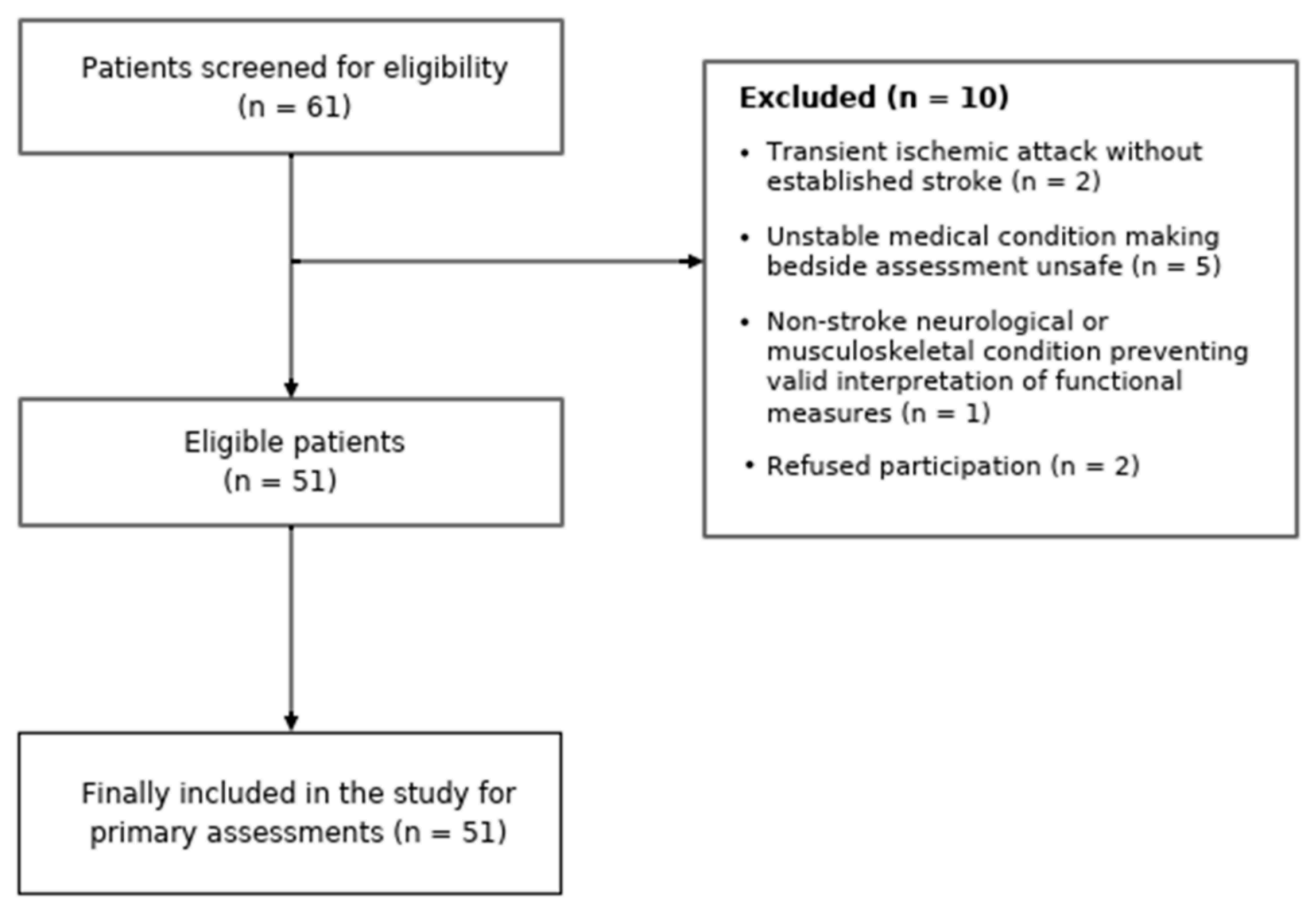
Flow diagram of participant recruitment and selection.

Our study population consisted of patients hospitalized with acute stroke. We recruited hospitalized patients with acute stroke using convenience sampling. Patients accessible to the study team during the recruitment period were screened for eligibility, and those meeting the inclusion criteria were invited to participate. Patients were included in the study if they were at least 18 years of age and above, had a clinical diagnosis of acute stroke established by neurologist and supported by the available clinical and neuroimaging findings, were hospitalized in the Clinic of Neurology, UCCK, were clinically stable enough to be able for the bedside assessment and were assessed within 24 h of admission to the participating clinical unit. Patients were excluded from the study if they had a transient ischemic attack without established stroke (2 patients), an unstable medical condition that made bedside assessment unsafe, such as hemodynamic instability, acute respiratory distress, or ongoing neurological deterioration requiring urgent treatment (5 patients); a non-stroke neurological or musculoskeletal condition that prevented valid interpretation of the primary functional measures (1 patient); or refused participation (2 patients) (Figure 1). We determined clinical stability before study assessment by considering the patient’s neurological, cardiovascular, and respiratory status. Patients were considered clinically stable when there was no ongoing clinical instability from above.

#### The Baseline Characteristics of Study Population

A total of 61 patients were screened for eligibility. Ten were excluded from the study: 2 patients had a transient ischemic attack without established stroke, 5 patients had an unstable medical condition that made bedside assessment unsafe, 1 patient had a non-stroke neurological or musculoskeletal condition that prevented valid interpretation of the primary functional measures, whilst 2 refused participation. Finally, a total of 51 patients were enrolled in the study. All participants had complete values for the PASS, component-derived FMA motor scores, Barthel Index, and modified Rankin Scale. All participants underwent assessment within 24 h of admission to the participating clinical unit, corresponding to 3–10 days after stroke onset. We established the sample size on a pragmatic basis based on how many eligible patients can be recruited and assessed within the defined study time period, finally resulting in a sample size of 51 subjects. No a priori sample size calculation or precision analysis was performed. Hence, the study sample represented feasible recruitment in this clinical setting. This study is exploratory in design, seeking to estimate relationships between postural control, limb motor impairment, and functional independence among hospitalized patients during acute stroke hospitalization, with added explanatory power from PASS. Effect sizes are reported along with 95% confidence intervals. Finally, the multivariable findings are interpreted as preliminary adjusted associations, whilst recommending confirmation in larger studies.

The mean age of all participants was 68.9 ± 12.3 years, with a median of 71 years (IQR 63–76), and 26 participants (51.0%) were male. Overall, 44 (86%) patients had ischemic stroke and a first-ever stroke [45, (88.2%)]. The affected hemisphere and clinically affected side were nearly evenly distributed. Spasticity was documented in 3 of 51 patients (5.9%) following assessment of muscle tone at the elbow and ankle using the Modified Ashworth Scale (MAS) (Table 1). Given the small number of affected patients, this finding was summarized descriptively, without separate analyses of its associations with postural control, motor function, or functional independence.

### 2.3. Definitions and Variables

For every patient, we administered a structured questionnaire through a face-to-face interview to collect demographic and relevant clinical information. The PASS, FMA, Barthel Index, and mRS assessments were administered by a multidisciplinary consisting of two neurologists, two physiatrists, and two certified physiotherapists. All assessors had more than 30 years of clinical experience and had received previous clinical training in the use of these assessment instruments. Assessors were blinded to the scores assigned by the other assessors. We performed the interview after the patient had been considered clinically stable and able to participate in the assessment. The time at which clinical stability was first established was not recorded separately; ergo, the interval from admission to clinical stability could not be quantified. Study assessments were performed within 24 h of admission to the participating clinical unit, at a mean of 5.7 ± 2.5 days after stroke onset (median 5 days; IQR 3–7 days). Provided that communication difficulties were present, we collected all the available information in the patient’s medical records or through a family member, when appropriate.

Following completion of the interview and review of the clinical record, each patient underwent a standardized bedside physical and functional assessment. As noted, we examined muscle tone using the MAS at the elbow and ankle, and we recorded for the presence of any detectable spasticity. Secondly, we evaluated the postural control using the Postural Assessment Scale for Stroke Patients (PASS), including both the maintaining-posture and changing-posture domains.

Thereafter, we assessed the motor impairment using the Fugl-Meyer Assessment (FMA). We evaluated upper-extremity and lower-extremity motor performance separately, allowing calculation of the FMA upper-extremity motor score, lower-extremity motor score, and overall motor score. We also assessed and recorded the additional FMA domains of sensation, passive range of motion, joint pain, and balance.

Second-to-last, after completion of the impairment- and postural-level assessments, we evaluated the patient’s ability to perform basic activities of daily living using the Barthel Index. Finally, overall disability was classified using the modified Rankin Scale (mRS). Baseline National Institutes of Health Stroke Scale (NIHSS) scores were unavailable in the study dataset and therefore could not be reported or included as an adjustment variable in the regression models.

#### 2.3.1. Postural Assessment Scale

We assessed the postural control using the Postural Assessment Scale for Stroke Patients (PASS), which is a 12-item scale specifically developed for individuals with stroke. Through PASS, we evaluated the ability to maintain and change posture, with each item scored from 0 to 3. The maintaining-posture subscale ranges from 0 to 15, the changing-posture subscale from 0 to 21, and the total PASS score from 0 to 36, with higher scores indicating better postural control.

#### 2.3.2. Fugl-Meyer Assessment

We evaluated both upper and lower limb motor impairment using the motor domain of the Fugl-Meyer Assessment (FMA). Following calculation of the overall FMA motor score, we also assessed and analyzed upper- and lower-extremity motor function using the FMA-UE and FMA-LE components. The upper-extremity motor score ranges from 0 to 66 and the lower-extremity motor score from 0 to 34. These components were summed to obtain a total motor score ranging from 0 to 100, with higher scores indicating better motor function and less severe impairment.

FMA motor score values <50 was classified as severe motor impairment, 50–84 as moderate impairment, 85–95 as mild impairment, 96–99 as very mild impairment or near-complete motor recovery, and 100 as normal motor function. The upper-extremity component was additionally interpreted separately: scores of 0–22 indicated severe impairment, 23–47 moderate impairment, 48–55 mild impairment, and 56–66 minimal impairment.

#### 2.3.3. Barthel Index

We assessed the functional independence in basic activities of daily living using the 10-item Barthel Index. The instrument evaluates feeding, bathing, grooming, dressing, bowel and bladder control, toilet use, transfers, mobility, and stair climbing. Scores range from 0 to 100, with higher values indicating greater functional independence. Clinicians determined Barthel Index scores using a combination of direct bedside observation, patient reports when communication and cognitive ability permitted, and information available in the medical records.

### 2.4. Statistical Analysis

Continuous variables were summarized as mean and standard deviation, whilst categorical variables as frequencies and percentages. We conducted a descriptive analysis for the entire study population expressed as mean arithmetic value with standard deviation, minimum and maximum value and total numbers.

We used Shapiro–Wilk test to assess the score distributions for normality. Spearman rank-correlation coefficients (ρ) were calculated to examine associations among PASS, FMA motor scores, Barthel Index, and modified Rankin Scale (mRS), with 95% confidence intervals (95% CI). We performed a multiple linear regression with the Barthel Index as the dependent variable to determine whether postural control was associated with functional independence beyond limb motor impairment. The selected predictors reflected the study’s explanatory objective, with age, assessment timing, and motor impairment forming the first adjustment block and PASS entered afterwards to estimate its additional contribution to Barthel Index variance, using continuous linear terms, and the change in R^2^ to quantify this contribution. In Model 1, age, days from stroke onset to assessment, and total FMA motor score were entered as covariates. Then, in Model 2, the PASS total score was added to assess whether postural control explained additional variance in Barthel Index scores after accounting for total FMA motor score, age, and assessment timing, as quantified by the change in R^2^ (ΔR^2^). To examine the relationship to upper-extremity motor function, we performed an additional regression analysis in which FMA-UE replaced the total FMA motor score. Model UE-1 included age, days from stroke onset to assessment, and FMA-UE, whilst PASS total score was subsequently added in Model UE-2. We included the time from stroke onset to assessment, expressed in days, as a continuous covariate in Models 1, 2, UE-1, and UE-2 to account for differences in assessment timing among participants. Each final main regression model included four predictor terms such as age, days from stroke onset to assessment, PASS, and either total FMA motor score or FMA-UE, plus an intercept. The two motor scores were evaluated in separate models. We also examined the Spearman correlation between the PASS total and FMA-UE. Hierarchical regression refers here to the sequential entry of predictor blocks into nested linear models, allowing the incremental variance explained by PASS (ΔR^2^) to be assessed after accounting for predictors. Results were reported as unstandardized regression coefficients (B), standardized coefficients (β), heteroscedasticity-consistent HC3 standard errors, 95% CI, and *p* values. We also assessed the multicollinearity using variance inflation factors (VIFs). We used ordinary least-squares regression to summarize adjusted associations on the Barthel Index point scale and quantify the additional variance explained by PASS. Residual diagnostics included Q–Q and residual-versus-fitted plots, the Shapiro–Wilk test of residual normality, and the Koenker–Breusch–Pagan test for heteroscedasticity. We performed fractional-logistic sensitivity analyses using Barthel Index scores divided by 100, retaining values of 0 and 1 and the same covariates as Models 2 and UE-2, with a logit link, Bernoulli quasi-maximum likelihood estimation, and HC3 robust standard errors to assess the consistency of the main associations.

For all statistical tests, a *p*-value of <0.05 was considered statistically significant. We treated PASS subanalyses, comparisons of FMA-UE and FMA-LE associations with the Barthel Index, and subgroup comparisons by sex, stroke type, and stroke history as secondary and exploratory, recognizing the increased risk of Type I error from multiple comparisons. Statistical data processing and analysis was performed using R (R version 4.5.2).

### 2.5. Ethical Considerations

The study was approved by the Ethics Committee of the Kosovo’s Doctors Chamber (Protocol number 1584) and Ethical Committee of Faculty of Medicine, University of Prishtina (Protocol number 1941). The study details were explained to the participants, verbally and in writing. Hence, all patients gave written informed consent, and the study was performed in accordance with the Helsinki Declaration.

## 3. Results

### 3.1. Postural, Motor, and Functional Scores and Their Associations with Functional Independence

The median PASS total score was 11 (IQR 3–25), and the median FMA motor total was 23 (IQR 11.5–82.5). Functional independence was generally low; the median Barthel Index was 15 (IQR 0–65), and 19 (37.3%) patients scored 0, whilst 5 (9.8%) scored 100. Overall disability was substantial, with a median modified Rankin Scale score of 5 (IQR 3.5–5); 32 (62.7%) patients were classified as mRS 5. FMA balance scores showed that 27 (52.9%) patients scored 0 (Table 2).

Both postural control and limb motor function were associated with functional independence. PASS total correlated with the Barthel Index at ρ = 0.932 (bootstrap 95% CI 0.866 to 0.965; *p* < 0.001), while FMA motor total correlated with the Barthel Index at ρ = 0.929 (95% CI 0.860 to 0.962; *p* < 0.001). The difference between these two dependent correlations was minimal (Δρ = 0.003, 95% CI −0.035 to 0.048; bootstrap *p* = 0.881), suggesting no evidence that either instrument had a stronger unadjusted association with the Barthel Index.

PASS and FMA motor total were also associated (ρ = 0.927, 95% CI 0.867 to 0.958; *p* < 0.001). PASS total was also strongly associated with upper-extremity motor performance measured using FMA-UE (ρ = 0.749, 95% CI 0.555 to 0.879; *p* < 0.001). Higher PASS, FMA motor, and Barthel scores were each associated with lower global disability on the modified Rankin Scale. The corresponding correlations were −0.858 for PASS, −0.842 for FMA motor total, and −0.888 for the Barthel Index, all significant for *p* < 0.001 (Table 3).

### 3.2. Regression Analysis of Adjusted Associations of Postural Control and Motor Impairment

In our first linear-regression model, age, days from stroke onset to assessment, and FMA motor total explained 90.2% of the variance in Barthel Index scores (adjusted R^2^ = 0.896). In this model, higher FMA motor scores had a strong association with higher Barthel scores (B = 0.926, 95% CI 0.835 to 1.017; *p* < 0.001). Older age showed a moderate negative association (B = −0.297, 95% CI −0.544 to −0.050; *p* = 0.018), while assessment timing was not associated with the outcome.

Then in second model we added PASS, which increased R^2^ from 0.902 to 0.950 (ΔR^2^ = 0.047; *p* < 0.001). In the final model, each one-point increase in PASS was associated with an estimated 1.615-point higher Barthel Index score (95% CI 0.975 to 2.254; standardized β = 0.527; *p* < 0.001), after accounting for age, assessment timing, and FMA motor impairment. FMA motor total also remained independently associated with the Barthel Index (B = 0.465, 95% CI 0.266 to 0.664; standardized β = 0.460; *p* < 0.001). Meanwhile, in the final model, age and stroke-assessment timing were not statistically significant (Table 4, Panel A).

### 3.3. Regression Analysis of Adjusted Associations of Postural Control and Motor Impairment for Upper Extremity

In the upper-extremity-specific linear-regression model, age, days from stroke onset to assessment, and FMA-UE explained 83.8% of the variance in Barthel Index scores (adjusted R^2^ = 0.828). In this model, higher FMA-UE scores had a strong association with higher Barthel Index scores (B = 1.196, 95% CI 1.024 to 1.369; *p* < 0.001). Older age showed a negative association with functional independence (B = −0.412, 95% CI −0.709 to −0.116; *p* = 0.006), while assessment timing was not associated with the outcome.

**Figure 2:**
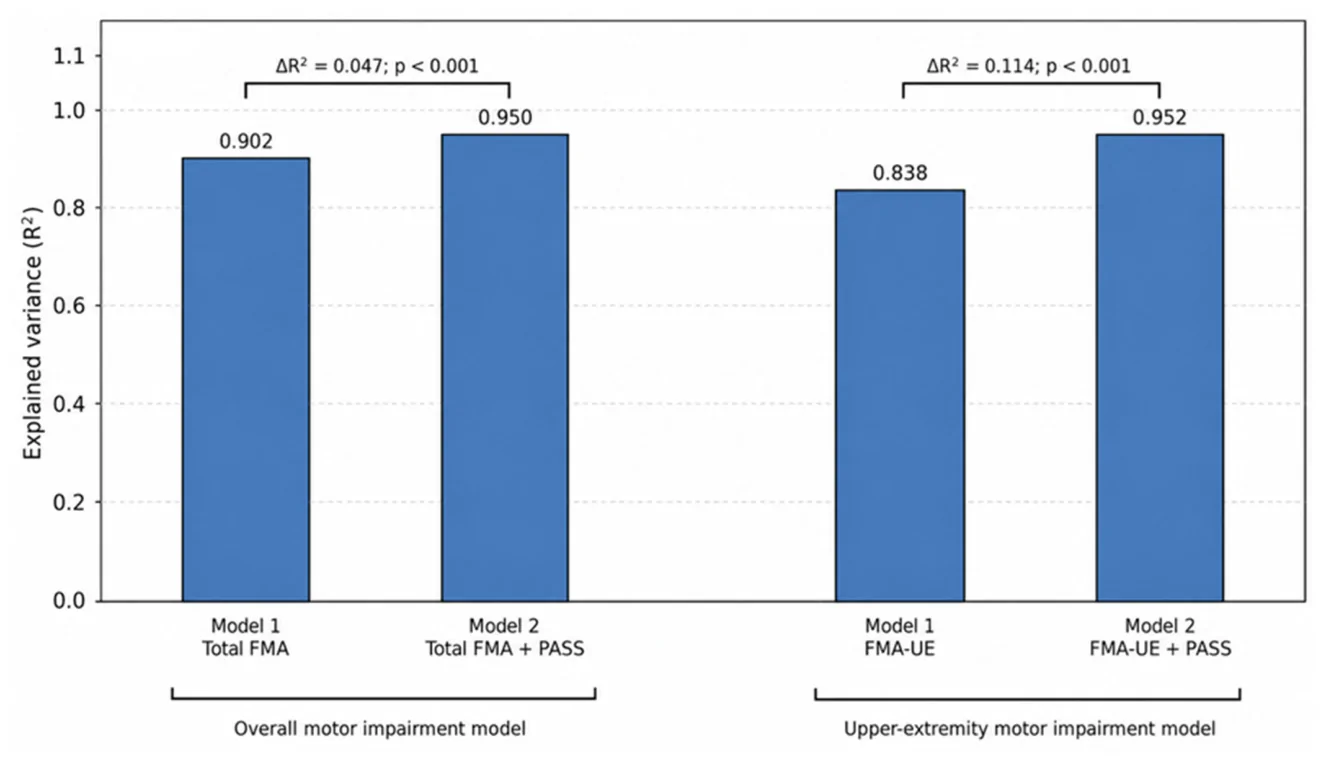
Incremental explanatory value of postural control beyond overall and upper-extremity motor impairment for functional independence.

In the second upper-extremity-focused model, PASS was added, increasing R^2^ from 0.838 to 0.952 (ΔR^2^ = 0.114; *p* < 0.001). In the final model, each one-point increase in PASS was associated with an estimated 1.955-point higher Barthel Index score (95% CI 1.469 to 2.441; standardized β = 0.638; *p* < 0.001), after accounting for age, assessment timing, and upper-extremity motor impairment. FMA-UE also remained independently associated with the Barthel Index (B = 0.505, 95% CI 0.296 to 0.713; standardized β = 0.368; *p* < 0.001). Meanwhile, age and stroke-assessment timing were no longer statistically significant (Table 4, Panel B, Figure 2).

Variance-inflation factors in the upper-extremity-focused final model were 3.29 for FMA-UE and 3.58 for PASS total, indicating moderate overlap between the two constructs. These values were lower than those observed in the overall motor model. A fractional-logistic sensitivity analysis for the bounded Barthel Index outcome confirmed the direction and statistical significance of both PASS and FMA-UE associations.

### 3.4. Associations of PASS Subdomains and Limb-Specific Motor Function with Functional Independence

Both PASS subdomains were strongly associated with the Barthel Index. The correlation was ρ = 0.943 (95% CI 0.887 to 0.973; *p* < 0.001) for maintaining posture and ρ = 0.929 (95% CI 0.863 to 0.961; *p* < 0.001) for changing posture. Although the maintaining-posture correlation was numerically higher, the difference between the two correlations was not statistically significant (Δρ = 0.014, 95% CI −0.013 to 0.045; *p* = 0.261). In separate adjusted models, both maintaining posture (B = 3.695, 95% CI 2.926 to 4.464; *p* < 0.001; R^2^ = 0.962) and changing posture (B = 2.229, 95% CI 0.576 to 3.881; *p* = 0.008; R^2^ = 0.932) remained associated with Barthel scores. Because the PASS subdomains were almost perfectly correlated with each other (ρ = 0.976), they were not entered simultaneously.

Lower-extremity motor function had a stronger unadjusted association with the Barthel Index than upper-extremity motor function. The FMA lower-extremity correlation was ρ = 0.915 (95% CI 0.829 to 0.957), compared with ρ = 0.799 (95% CI 0.619 to 0.907) for the upper extremity; the difference was 0.116 (95% CI 0.006 to 0.267; *p* = 0.037). This finding was considered exploratory. FMA balance correlated strongly with PASS total (ρ = 0.902), Barthel Index (ρ = 0.924), and modified Rankin Scale (ρ = −0.900), all *p* < 0.001 (Table 5).

We found no statistically detectable differences in PASS, FMA motor, Barthel Index, or modified Rankin Scale scores were observed by sex, stroke history, or ischemic versus hemorrhagic/hemorrhagic-transformation grouping. These comparisons were exploratory and underpowered because only seven patients were included in the combined hemorrhagic group and six had recurrent stroke (Supplementary Table S1). These comparisons were exploratory, and the small combined hemorrhagic group and recurrent-stroke group may have limited our ability to detect differences.

## 4. Discussion

### 4.1. Main Findings

In our study which is the first of its kind in the region to date and to the best of our knowledge, we found that postural control and motor impairment were associated with functional independence and global disability during acute stroke hospitalization. We assessed patients at a median of 5 days after onset and when they showed substantial functional impairment and dependency, with a median Barthel Index of 15 and almost two-thirds classified as mRS 5. PASS and total FMA motor scores showed similar unadjusted associations with the Barthel Index. Adding PASS to the model containing age, timing, and total FMA motor score increased explained variance from 90.2% to 95.0% (ΔR^2^ = 0.047; *p* < 0.001). These findings align with earlier studies linking motor impairment, balance, and activities of daily living [9,13], and contemporary evidence supporting the functional importance of postural and trunk control [10,11,16,21,22,23,24,25].

In the upper-extremity-focused analysis we found that FMA-UE was strongly associated with both PASS and functional independence. Adding PASS to the model containing age, assessment timing, and FMA-UE increased explained variance from 83.8% to 95.2% (ΔR^2^ = 0.114; *p* < 0.001). Both FMA-UE and PASS remained significantly associated with the Barthel Index, suggesting that postural control provided additional information beyond upper-extremity motor impairment. Our findings extend previous evidence linking trunk control, upper-extremity function, and daily activities during early stroke recovery [11,16,22,24,25], while remaining consistent with foundational studies of trunk performance and functional outcomes [12,13].

Age was associated with lower Barthel Index scores before PASS was added but was no longer significant in the final model. We also found that the assessment timing was not independently associated with functional independence. Despite substantial clustering at the lower end of the Barthel Index, fractional-logistic sensitivity analysis supported the direction and statistical significance of the associations of PASS and FMA motor scores with functional independence.

The findings were also aligned when we considered the bounded distribution of the Barthel Index. Despite substantial clustering at the lower end of the scale, fractional-logistic sensitivity analysis confirmed the direction and statistical significance of the associations of both PASS and FMA motor scores with functional independence. This consistency reduces the likelihood that the principal findings were driven solely by the distributional characteristics of the Barthel Index.

### 4.2. Contextual Comparison with Current Evidence and Clinical Relevance

The relationship between postural control and upper-extremity function was clinically plausible, as trunk stability supports positioning, reaching, and controlled arm movement. Studies show that upper-extremity recovery remains an important component of functional restoration after stroke [26], while recent studies have linked trunk control with upper-extremity performance and examined the contribution of trunk-focused rehabilitation to upper-limb and activity-level outcomes [10,11,16,22,23,24,25].

Simultaneously, PASS and FMA assess related but distinct dimensions of post-stroke function. FMA characterizes limb motor impairment, whereas PASS evaluates the ability to maintain and change posture. Studies demonstrated relationships among FMA motor performance, balance, and Barthel-based independence [9], while recent evidence supports the interaction among trunk control, limb function, and daily activities [10,11,16,21,22,23,24,25]. Although strongly associated in our study, these measures should not be considered interchangeable.

In our study, both PASS subdomains remained associated with the Barthel Index in separate adjusted analyses. Maintaining posture showed a numerically stronger association, yet the difference was not statistically significant. These findings align with current evidence supporting the clinical utility of PASS [5,27,28]. However, the almost perfect correlation between subdomains prevents clear separation of their contributions, and these findings remain exploratory.

Lower-extremity FMA performance showed a stronger unadjusted association with functional independence than upper-extremity performance, possibly reflecting the mobility-related content of the Barthel Index. Previous studies linked lower-extremity motor performance with balance and mobility [8,9]. Nevertheless, the upper-extremity function remained strongly associated with independence and is essential for self-care. Studies with larger study population and multiple study centers are recommended. Finally, we could not determine the contribution of spasticity to the functional limitations observed in this study. Repeated assessments during follow-up would help characterize its subsequent development and functional relevance, consistent with recommendations for regular spasticity assessment throughout stroke care [29].

From the findings in our study, the distribution of balance scores were also noteworthy. More than half of the participants scored 0 on the FMA balance component, whereas none reached the minimum PASS total score. PASS was developed to assess a broad range of stroke severity and has demonstrated validity during acute and early recovery stages [4,5,27,28]. Previous evidence suggests that PASS may be less affected by lower-end measurement limitations [28]. The clustering of FMA balance scores at zero may indicate limited discrimination among severely impaired patients, although our study was not designed as a formal psychometric comparison and does not establish PASS superiority.

In our study, PASS, FMA motor, and Barthel Index scores were strongly inversely associated with global disability on the mRS, with the strongest relationship between the Barthel Index and mRS. This aligns with previous evidence showing substantial correspondence between Barthel-based independence and mRS grades [30,31]. Although both measure disability and dependency, the Barthel Index assesses specific activities of daily living, whereas the mRS provides a broader ordinal classification. The strong associations of PASS and FMA with mRS further support the relationship between postural and motor impairment and overall disability during acute stroke.

The additional contribution of PASS is clinically plausible because independence in basic activities requires not only voluntary limb movement but also postural stability and safe transitions between positions. Sitting, standing, shifting body position, transfers, and mobility all require coordinated trunk and postural control. Conversely, postural control cannot fully compensate for severe limb motor impairment. The persistence of both PASS and FMA motor scores in the final model therefore supports a multidimensional approach to functional assessment during acute stroke hospitalization.

The strong overlap between PASS and total FMA motor score should nevertheless be considered when interpreting the regression results. Meanwhile, the upper-extremity-specific model showed less multicollinearity. Nevertheless, these measures remain related components of post-stroke motor function, and their coefficients should therefore be interpreted as adjusted associations rather than completely independent biological effects. VIFs were 5.47 for total FMA motor score and 5.87 for PASS in the overall motor model (Model 2), compared with 3.29 for FMA-UE and 3.58 for PASS in the upper-extremity model (Model UE-2). Rather, two highly related constructs each retained statistically detectable information associated with functional independence. Likewise, the additional 4.7% of variance explained by PASS represents incremental explanatory information within this sample, not evidence of a causal effect, which cannot be established from the cross-sectional design. The increases in R^2^ after adding PASS represent incremental explanatory information conditional on age, assessment timing, and the motor score included in each model.

The study provides data from Kosovo, where published evidence on standardized functional assessment during acute stroke hospitalization remains limited. To date and to the best of our knowledge, this is the first study of this nature in Kosovo.

### 4.3. Strengths and Limitations

Our study had several strengths. Firstly, we assessed postural control, upper- and lower-limb motor impairment, functional independence, and global disability within the same acute-stroke evaluation using established clinical instruments. Secondly, we moved beyond simple correlations and tested whether PASS contributed additional explanatory information after accounting for overall FMA motor impairment, age, and assessment timing. Also, several limitations should be recognized. Firstly, the cross-sectional and single-center design prevents causal or prognostic conclusions and may limit generalizability. Secondly, the relatively moderate sample size of 51 patients, restricted subgroup analyses, particularly for hemorrhagic and recurrent stroke, limiting the precision and stability of multivariable estimates and potentially increasing overfitting, while particularly restricting analyses of hemorrhagic and recurrent stroke. Thirdly, we did not have NIHSS scores, detailed lesion characteristics, or longitudinal follow-up, which may leave residual confounding and prevents assessment of later recovery. Finally, the strong correlation between PASS and FMA motor scores indicates considerable overlap between these constructs, so their adjusted regression effects should be interpreted with caution. Additional limitations include the absence of an a priori sample-size calculation, possible selection bias from convenience sampling, possible information bias from family reports, and lack of adjustment for rehabilitation intensity before assessment. Their coefficients should rather be interpreted as adjusted associations between overlapping functional domains, without implying distinct causal effects.

## 5. Conclusions

Our findings suggest that postural control and limb motor impairment were strongly associated with functional independence during acute stroke hospitalization. PASS added explanatory information within this study sample, but the substantial overlap among the measures warrants caution when interpreting their distinct contributions to functional independence. These findings add evidence from Kosovo supporting a multidimensional approach to acute stroke assessment. We highlight the need for larger multicenter longitudinal studies to validate and determine whether combining these measures improves prediction of subsequent functional recovery and rehabilitation outcomes.

## Figures and Tables

**Table 1:** Demographic and clinical characteristics of the study patients, Clinic of Neurology, University Clinical Centre of Kosova (UCCK), April–July, 2026 (n = 51).

	Characteristic	Value
**Age, years**		68.9 ± 12.3; 71 [63–76]
**Days from stroke onset to assessment**		5.7 ± 2.5; 5 [3–7]
**Sex**	Male	26 (51.0)
	Female	25 (49.0)
**Stroke type**	Ischemic	44 (86.3)
	Hemorrhagic	3 (5.9)
	Ischemic with hemorrhagic transformation	4 (7.8)
	Stroke history	
	First-ever stroke	45 (88.2)
	Recurrent stroke	6 (11.8)
**Affected cerebral hemisphere**	Left	25 (49.0)
	Right	26 (51.0)
**Clinically affected side**	Right	25 (49.0)
	Left	26 (51.0)
**Spasticity present**		3 (5.9)

**Table 2:** Distribution of postural, motor, and functional assessment scores, Clinic of Neurology, University Clinical Centre of Kosova (UCCK), April–July, 2026.

Measure	Possible Range	Mean ± SD	Median [IQR]	Observed Range	Minimum Score, n (%)	Maximum Score, n (%)
**PASS maintaining posture**	0–15	4.6 ± 5.0	3 [0–9]	0–15	15 (29.4)	6 (11.8)
**PASS changing posture**	0–21	9.6 ± 6.9	8 [3–16.5]	2–21	0 (0.0)	2 (3.9)
**PASS total**	0–36	14.2 ± 11.8	11 [3–25]	2–36	0 (0.0)	2 (3.9)
**FMA upper-extremity motor**	0–66	25.8 ± 26.3	6 [4–59]	0–66	2 (3.9)	1 (2.0)
**FMA lower-extremity motor**	0–34	16.1 ± 10.7	14 [8–26.5]	2–34	0 (0.0)	3 (5.9)
**FMA motor total**	0–100	41.9 ± 35.7	23 [11.5–82.5]	3–100	0 (0.0)	1 (2.0)
**Barthel Index**	0–100	30.5 ± 36.0	15 [0–65]	0–100	19 (37.3)	5 (9.8)
**Modified Rankin Scale**	0–6	4.1 ± 1.3	5 [3.5–5]	1–5	0 (0.0)	32 (62.7)
**FMA balance**	0–14	4.3 ± 5.1	0 [0–8]	0–14	27 (52.9)	6 (11.8)

**Table 3:** Spearman correlations among postural control, motor impairment, functional independence, and global disability.

Association	Spearman ρ	95% CI	*p*
**PASS total-Barthel Index**	0.932	0.866 to 0.965	<0.001
**FMA motor total-Barthel Index**	0.929	0.860 to 0.962	<0.001
**PASS total-FMA motor total**	0.927	0.867 to 0.958	<0.001
**PASS total-modified Rankin Scale**	−0.858	−0.917 to −0.748	<0.001
**FMA motor total-modified Rankin Scale**	−0.842	−0.913 to −0.727	<0.001
**Barthel Index-modified Rankin Scale**	−0.888	−0.947 to −0.786	<0.001
**PASS total-FMA-UE**	0.749	0.555 to 0.879	<0.001

**Table 4:** Regression models of adjusted associations of postural control and motor impairment.

**Panel A. Overall Motor Impairment Model**						
**Model**	**Predictor**	**B**	**HC3 SE**	**95% CI**	**Standardized β**	***p***
**Model 1**	Age	−0.297	0.126	−0.544 to −0.050	−0.101	0.018
	Days since onset	0.532	0.660	−0.761 to 1.826	0.037	0.420
	FMA motor total	0.926	0.046	0.835 to 1.017	0.917	<0.001
**Model 2**	Age	−0.086	0.101	−0.285 to 0.113	−0.029	0.397
	Days since onset	0.407	0.569	−0.707 to 1.522	0.028	0.474
	FMA motor total	0.465	0.102	0.266 to 0.664	0.460	<0.001
	PASS total	1.615	0.326	0.975 to 2.254	0.527	<0.001
**Panel B. Upper-extremity motor impairment model**						
**Model**	**Predictor**	**B**	**HC3 SE**	**95% CI**	**Standardized β**	***p***
**UE Model 1**	Age	−0.412	0.151	−0.709 to −0.116	−0.141	0.006
	Days since onset	0.398	0.830	−1.230 to 2.025	0.028	0.632
	FMA-UE	1.196	0.088	1.024 to 1.369	0.873	<0.001
**UE Model 2**	Age	−0.074	0.097	−0.263 to 0.115	−0.025	0.444
	Days since onset	0.315	0.551	−0.765 to 1.395	0.022	0.568
	FMA-UE	0.505	0.106	0.296 to 0.713	0.368	<0.001
	PASS total	1.955	0.248	1.469 to 2.441	0.638	<0.001

Model UE-1: R^2^ = 0.838, adjusted R^2^ = 0.828. Model UE-2: R^2^ = 0.952, adjusted R^2^ = 0.948; ΔR^2^ = 0.114, *p* < 0.001.

**Table 5:** Secondary analysis of associations of PASS subdomains and limb-specific motor function with functional independence.

Modeled Variables	Estimate	95% CI	*p*
**PASS maintaining posture-Barthel Index**	ρ = 0.943	0.887 to 0.973	<0.001
**PASS changing posture-Barthel Index**	ρ = 0.929	0.863 to 0.961	<0.001
**Difference between PASS subscale correlations**	Δρ = 0.014	−0.013 to 0.045	0.261
**Maintaining-posture adjusted model**	B = 3.695; R^2^ = 0.962	2.926 to 4.464	<0.001
**Changing-posture adjusted model**	B = 2.229; R^2^ = 0.932	0.576 to 3.881	0.008
**FMA upper-extremity motor-Barthel Index**	ρ = 0.799	0.619 to 0.907	<0.001
**FMA lower-extremity motor-Barthel Index**	ρ = 0.915	0.829 to 0.957	<0.001
**Difference: lower- minus upper-extremity correlation**	Δρ = 0.116	0.006 to 0.267	0.037
**FMA balance-PASS total**	ρ = 0.902	-	<0.001
**FMA balance-Barthel Index**	ρ = 0.924	-	<0.001
**FMA balance-modified Rankin Scale**	ρ = −0.900	-	<0.001

## Data Availability

The data presented in this study are available on request from the corresponding author, based on the Ethical Approval Article of Patient Confidentiality.

## Supplementary Materials

The following supporting information can be downloaded at https://www.mdpi.com/article/10.3390/healthcare14203362/s1. Table S1: Associations and group comparisons of PASS subdomains and limb-specific motor function with functional independence.
