# Supplementary material for: Postural Control and Upper-Extremity Motor Impairment Associated with Functional Independence During Acute Stroke Hospitalization: A Cross-Sectional Study
Source: Healthcare (Basel). 2026 Oct 9;14(20):3362. doi: 10.3390/healthcare14203362 (PMC13704710; doi:10.3390/healthcare14203362)
Supplement: Supplementary file 1 [file healthcare-14-03362-s001.zip › healthcare-4546880-supplementary.pdf]

Supplementary Table S1. Associations and group comparisons of PASS subdomains and limb-specific motor function with functional independence

Grouped variables	Outcome	Group 1 median	Group 2 median	p	Cliff's δ*
**Stroke type: ischemic vs hemorrhagic/HT**	PASS total	11.5	8.0	0.826	0.055
	FMA motor total	25.5	16.0	0.945	0.019
	Barthel Index	15.0	0.0	0.673	0.101
	Modified Rankin Scale	5.0	5.0	0.764	0.065
**Stroke history: first-ever vs recurrent**	PASS total	11.0	12.0	1.000	0.000
	FMA motor total	23.0	23.0	0.872	0.044
	Barthel Index	15.0	17.5	0.952	-0.019
	Modified Rankin Scale	5.0	5.0	0.774	-0.067
**Sex: male vs female**	PASS total	7.5	13.0	0.378	-0.145
	FMA motor total	17.0	34.0	0.485	-0.115
	Barthel Index	5.0	25.0	0.233	-0.191
	Modified Rankin Scale	5.0	5.0	0.433	0.112

**Cliff's δ represents the non-parametric effect size for between-group differences; values closer to 0 indicate little or no difference, while values approaching ±1 indicate greater separation between groups.*
